# Cardiovascular Risk of Concomitant Use of Atypical Antipsychotics and Stimulants Among Commercially Insured Youth in the United States

**DOI:** 10.3389/fpsyt.2021.640244

**Published:** 2021-04-29

**Authors:** Chengchen Zhang, O'Mareen Spence, Gloria Reeves, Susan DosReis

**Affiliations:** ^1^Department of Pharmaceutical Health Services Research, University of Maryland School of Pharmacy, Baltimore, MD, United States; ^2^Division of Child and Adolescent Psychiatry, University of Maryland School of Medicine, Baltimore, MD, United States

**Keywords:** youth, atypical antipsychotics, stimulants, cardiovascular risk, drug safety

## Abstract

**Objectives:** To investigate the risk of cardiovascular events associated with concomitant use of stimulants and atypical antipsychotics (AAPs) among youth and evaluate whether AAP dose and duration of concomitant use modifies the risk.

**Methods:** We used IQVIA PharMetrics® Plus data from 2006 to 2015 to construct a retrospective cohort of commercially-insured youth aged 5–17 years old who initiated a stimulant medication. Time-varying concomitant stimulant/AAP use was defined as current, past and no concomitant use based on person months. The primary time-varying Cox proportional hazard regression analysis evaluated the risk of cardiovascular events comparing current concomitant use with past and no concomitant use, adjusted for baseline cardiovascular risk. A secondary analysis assessed the risk of cardiovascular events comparing AAP daily doses (<1, 1–2, >2 mg) and duration (<3, 3–6, >6 months) of current concomitant use to no concomitant use. Cardiovascular outcomes included severe (i.e., stroke, acute myocardial infarction, ischemic heart disease) and less severe (i.e., angina pectoris, cardiac dysrhythmias, transient cerebral ischemia, hypertensive disease, tachycardia, palpitations, syncope).

**Results:** For this cohort of 61,438 youths, the incidence rate of severe cardiovascular events was 0.18 per 10,000 person-months, and all events occurred in no concomitant use months. The risk of less severe cardiovascular events was significantly higher in current concomitant users compared with no [HR: 2.59 (95%CI: 1.72, 3.90)] and past [HR: 1.89 (95%CI: 1.10, 3.24)] concomitant users. Compared to no concomitant use, the risk of less severe cardiovascular events was significantly higher at all AAP daily doses [HR: <1 mg: 2.82 (95%CI: 1.72, 4.61); 1–2 mg: 2.22 (95%CI: 1.16, 4.25); >2 mg: 2.65 (95%CI: 1.50, 4.71)]. The risk of less severe cardiovascular events significantly elevated for all duration of use and was higher for <3 months of concomitant use [HR: <3 months: 3.45 (95%CI: 2.17, 5.47) relative to 3–6 months: 2.60 (95%CI: 1.29, 5.25) or >6 months: 2.61 (95%CI: 1.59, 4.30)].

**Conclusions:** Severe cardiovascular events are rare. Concomitant stimulant/AAP use elevates the risk of less severe cardiovascular events. Periodic heart rate or blood pressure monitoring for youth on stimulant/AAP treatment may be warranted.

## Introduction

Stimulants are considered the first-line pharmacological treatment for Attention-Deficit/Hyperactivity Disorder (ADHD), and are widely used among youth in the U.S. (1). While the efficacy of stimulants for ADHD is well-supported (2, 3), the cardiovascular safety of stimulants has been equivocal. Several large population-based studies have not found a significant elevated risk of serious cardiovascular events, including stroke, myocardial infarction, and cardiac sudden death, related to stimulant use among youth (4, 5). But other studies have reported an increased risk of cardiac-related hospitalization and emergency department visits associated with stimulant use among youth and young adults (6, 7). The current US Food and Drug Administration (FDA) labeling warns against prescribing stimulants to patients with serious heart problems and recommends periodic heart rate or blood pressure monitoring among youth prescribed stimulants (8).

The majority of the evidence for the cardiovascular safety of pediatric stimulant use does not account for the concomitant use of stimulants with other psychotropic classes, such as atypical antipsychotics (AAPs), which happens after initiating the simulant treatment among some children and adolescents (9). AAPs are FDA approved for the treatment of pediatric schizophrenia, bipolar disorder, irritability associated with autism, and Tourettes' disorder, but are also commonly used for non-approved purposes (i.e., off-label use), such as managing behavioral symptoms (10–12). The concomitant use of AAPs with stimulants presents potential cardiac safety concerns because AAP use in youth is associated with increased cardiovascular events (13). Concomitant use of medications from multiple psychotropic classes is known to produce adverse drug reactions that can be additive (14, 15), but there is limited evidence for or against the cardiovascular effects of concomitant stimulant and AAP use beyond possible drug-drug interactions (16). Given recent increases in the use of AAPs concomitantly with stimulants among US youth (17–19), the scarcity of research that has examined the cardiovascular safety with such concomitant use represents a significant evidence gap. The primary objective of this study was to investigate the cardiovascular risk of concomitant stimulant and AAP use in a large retrospective cohort of commercially-insured youth in the US. Since prior studies suggest that risk of cardiovascular events can be associated with dose of AAP (13), a secondary objective investigated whether AAP dose and duration of concomitant use moderate cardiovascular risk among youth. The study was approved by the University *[blinded for review]* Institutional Review Board.

## Methods

### Study Design

A new user retrospective cohort was constructed among commercially-insured US youth. A cohort of youth who newly initiated a stimulant medication and had no baseline AAP use was selected.

### Study Cohort

**Figure 1:**
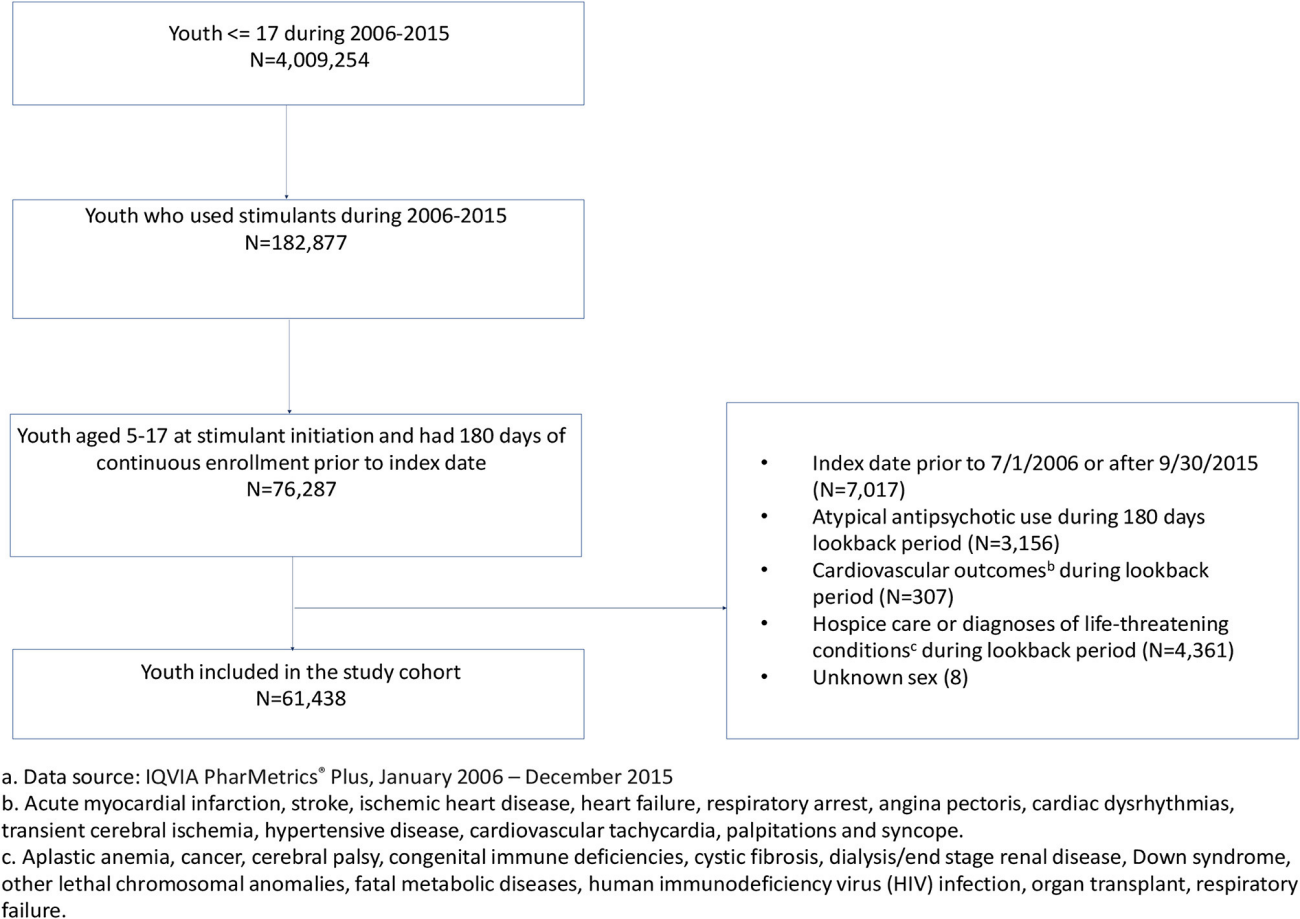
Cohort identification of commercially-insured youth (5–17 years old) who were new users of stimulants, 2006–2015.^a^ ^a^Data source: IQVIA PharMetrics® Plus, January 2006–December 2015. ^b^Acute myocardial infarction, stroke, ischemic heart disease, heart failure, respiratory arrest, angina pectoris, cardiac dysrhythmias, transient cerebral ischemia, hypertensive disease, cardiovascular tachycardia, palpitations and syncope. ^c^Aplastic anemia, cancer, cerebral palsy, congenital immune deficiencies, cystic fibrosis, dialysis/end stage renal disease, Down syndrome, other lethal chromosomal anomalies, fatal metabolic diseases, human immunodeficiency virus (HIV) infection, organ transplant, respiratory failure.

The study cohort comprised youth aged 5–17 years old at the time of their first stimulant prescription identified in the data between July 1 2006 and September 30 2015. The date of the first stimulant prescription defined the index date. To be included in the cohort, youth were further required to be continuously enrolled in their healthcare insurance for at least 180 days prior to the index date and have no AAP prescriptions or cardiovascular events of interest during the 180-day look-back period. We further excluded youth with serious medical conditions related to a high risk of developing cardiovascular outcomes. These conditions included aplastic anemia, cancer, cerebral palsy, congenital immune deficiencies, cystic fibrosis, dialysis/end stage renal disease, Down syndrome, other lethal chromosomal anomalies, fatal metabolic diseases, human immunodeficiency virus (HIV) infection, organ transplant, respiratory failure or receipt of hospice care (1, 4, 20). Cohort selection is shown in Figure 1.

### Data Source

We used a 10% sample of IQVIA PharMetrics® Plus data from 2006 through 2015. IQVIA PharMetrics® Plus data contains fully adjudicated medical and pharmacy claims and is generally representative of the commercially insured population in the US. The data provides de-identified person-level information including year of birth, sex, and monthly enrollment in medical and pharmacy benefits and claim-level information for medical service use and pharmacy dispensings. The medical service use represents inpatient, outpatient and emergency department visits, which contain information on clinical diagnoses recorded as the International Classification of Diseases, Ninth Revision, Clinical Modification (ICD-9-CM) codes and on procedures performed during the visit recorded with the Current Procedural Terminology (CPT-4) codes or the Healthcare Common Procedure Coding System (HCPCS) codes. Pharmacy data, which represent prescriptions filled at outpatient pharmacies, include a unique National Drug Code (NDC) that specifies the drug name and strength, a generic product identifier (GPI) that identifies the therapeutic classification, the dispensing date, the quantity dispensed, and the days supplied.

### Stimulant and Antipsychotic Exposure Measures

#### Stimulant and AAP Use

Stimulants and AAPs were identified from outpatient pharmacy claims data. Stimulants included methylphenidate and mixed amphetamine salts. AAPs included aripiprazole, olanzapine, clozapine, quetiapine, paliperidone, risperidone, ziprasidone, asenapine, and iloperidone. Using the date of dispensing and the days supplied of the medication, we determined whether each day of follow-up was a stimulant use day, an AAP use day, or no stimulant or AAP use day. A day that was both stimulant and AAP use was defined as a concomitant stimulant/AAP use day. We allowed a 30-day lag to account for the carry-over effect of AAPs. Carry-over effect refers to the effect that continues after the treatment ceases and is applied in previous studies of AAPs (13, 21, 22). AAP use days were classified as a no AAP use day when the prescription was discontinued for 30 days or more. No lag was applied to classify stimulant use days since no carryover effects of stimulants are reported in the literature (23, 24).

#### Concomitant Use of Stimulants and AAPs

**Figure 2:**
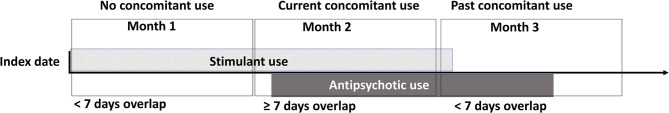
Illustration of time-varying definition of concomitant stimulant/AAP use.

Figure 2 provides a sample to illustrate the definition of time-varying concomitant stimulant/AAP use. Exposure to concomitant stimulant/AAP use was defined in a time-varying manner based on follow-up months. As the cohort was nested in youth who initiated a stimulant medication and had no previous AAP use, all follow-up time started as “no concomitant use.” A month of “current concomitant use” was defined as a month with 7 days or more of overlapping use of stimulant and AAP. A month with <7 days of overlapping stimulant/AAP use that followed a period of current concomitant use was categorized as “past concomitant use.” A youth was allowed to switch back and forth between current and past concomitant use.

#### AAP Dose and Duration of Concomitant Use

To calculate the average daily AAP dose for each follow-up month, we multiplied the strength of AAP by the quantity dispensed in the 30-day month divided by 30. We used an established formula (13) to convert the average AAP daily dose to risperidone equivalents to permit dose comparisons across individual agents. The average daily AAP dose was classified into three categories: <1, 1–2, and >2 mg.

Concomitant stimulant/AAP use duration was a time-varying measure of the cumulative number of current concomitant use months since the index date. The duration of concomitant use was classified into three categories: <3, 3–6, and >6 months of use.

### Cardiovascular Outcomes

Our study outcome was guided by prior research that documented the association between stimulants and serious cardiovascular events (i.e., stroke, myocardial infarction, ischemic heart disease), and cardiovascular symptoms (i.e., cardiac dysrhythmias, tachycardia, palpitations) (1, 4, 6, 8, 13). Therefore, we assessed two composite cardiovascular outcomes in this study: (1) severe cardiovascular events and (2) less severe cardiovascular events. Severe cardiovascular events were defined as an incident inpatient or emergency department (ED) visit claim with a primary or secondary diagnosis of acute myocardial infarction (ICD-9-CM:410), stroke (ICD-9-CM: 430, 431, 433, 434, and 436), ischemic heart disease (ICD-9-CM: 411.89), heart failure (ICD-9-CM: 428) or respiratory arrest (ICD-9-CM: 799.1). The codes for stroke, acute myocardial infarction, and ischemic heart disease are validated in adults (25–29) and acute myocardial infarction and stroke are also validated in youth (4). All codes listed for severe cardiovascular events have been applied in previous studies to define severe cardiovascular events among youth (1, 13). Less severe cardiovascular events were defined as an incident inpatient or ED visit claim with a primary or secondary diagnosis of angina pectoris (ICD-9-CM: 413), cardiac dysrhythmias (ICD-9-CM:427), transient cerebral ischemia (ICD-9-CM: 435), hypertensive disease (ICD-9-CM:401-405), cardiovascular tachycardia (ICD-9-CM: 785.0), palpitations (ICD-9-CM:785.1) or syncope (ICD-9-CM: 780.2), or two incident consecutive outpatient visits of cardiac dysrhythmia or palpitations within 14 days. The listed codes for less severe cardiovascular events have been utilized in prior studies to identify cardiovascular symptoms among youth taking psychotropic medications (1, 6, 13, 30, 31).

### Baseline Covariates

The 180-day lookback period defined the baseline period. Covariates examined during the baseline period included age, sex, and psychiatric comorbidities (i.e., ADHD, schizophrenia, development disorders, Tic disorder, bipolar disorder, disruptive behavior disorder, depressive disorder, anxiety disorder, adjustment disorder, communication and learning disorder, alcohol and other substance and other psychiatric disorders). To generate real-world evidence of actual clinical practice and to follow methods used other studies in order to maintain consistency with and comparison to prior research, we assessed baseline cardiovascular disorders which were not defined as study outcomes (e.g., abnormal heart sound, cardiac shock, etc.), respiratory conditions (e.g., asthma, bronchitis, etc.), use of medications to treat cardiovascular disease or other predisposing conditions (e.g. ACE inhibitors, cardiac-selective β blockers, antiarrhythmics, etc.) or metabolic conditions (e.g., diabetes, hyperlipidemia, thyroid related disorders, etc.), and use of contraceptive medications and devices (1, 13, 20). Likewise, we assessed congenital anomalies of the heart and circulatory system at baseline because youth with these anomalies are vulnerable to adverse cardiac effects of medications in concert with prior research (4). The covariates were identified based on ICD-9-CM diagnosis codes, CPT-4 procedure codes, and generic drug names. The generic product identifier was also used to identify cardiovascular medications and anxiolytics. The full list of baseline covariates is in Appendix A in Supplementary Material.

### Statistical Analysis

The descriptive analysis of baseline characteristics included frequencies and proportions for categorical variables, and medians and inter-quartile range (IQR) for continuous variables. The cohort was characterized by age group (5–9, 10–14, 15–17), sex, US region of residence, psychotropic use, psychiatric comorbidities, and length of follow-up in the study.

Time-dependent Cox proportional hazard regression models were used to estimate the risk of cardiovascular events accounting for time-varying exposure to concomitant stimulant/AAP use. The unit of analysis was person-month. Youth were followed from the index date until they experienced either a severe or less severe cardiovascular event or were censored (whichever came the first). For those with repeated cardiovascular events, only the first occurrence was counted. Censoring events included the development of an aforementioned serious medical condition identified as exclusion criteria, stimulant discontinuation, age 21, loss to follow-up, or the end of the study (September 30, 2015). Stimulant discontinuation was defined as no stimulant prescriptions for six or more consecutive months during the follow-up. Youth were considered loss to follow-up if they lost the health insurance coverage for six or more consecutive months. To evaluate the potential impact of missingness due to uninsured months on the risk estimate and the interpretation of our finding, we conducted a sensitivity analysis allowing no more than 1 month of loss of health insurance coverage during follow-up.

We constructed a disease risk score (DRS) using Miettinen full-cohort approach to adjust for confounding (32, 33). DRS is a summary score to describe the probability of developing the outcome as a function of baseline covariates. Unlike the propensity score that models the likelihood of receiving treatment, the DRS balances confounders of the underlying risk to develop the outcome. Full-cohort DRS performs similarly to a propensity score but reduces the computational complexity of fitting models with multiple time-varying exposures (34–36). Using a logistic regression model, the DRS was developed for a composite outcome, consisting of all cardiovascular events included in the study, in which baseline covariates were independent variables. The constructed DRS was categorized into tertile ranks and included as a covariate in the final time-varying Cox proportional hazard regression models.

We estimated the risk of severe and less severe cardiovascular events in separate regression models. The primary Cox regression model estimated the hazard ratios (HRs) of cardiovascular events comparing current with no concomitant use, past with no concomitant use, and current with past concomitant use, adjusted for average AAP daily dose, duration of concomitant use and DRS. The secondary Cox regression model estimated the HRs of cardiovascular events for average AAP daily doses (<1, 1–2, >2 mg) and duration (<3, 3–6, >6 months) of current concomitant use comparing with no concomitant use, adjusted for DRS.

## Results

### Baseline Characteristics of the Cohort

There were 61,438 youth who were new stimulant users, among whom 67.8% initiated with methylphenidate and 32.2% initiated with mixed amphetamine salts. The median length of follow-up was 11 months (IQR: 20 months). The majority of youth were male (68.2%), and aged 10 to 17 years old (59.2%). During baseline, the leading psychotropic medication use, in addition to stimulants, included selective serotonin reuptake inhibitors (SSRIs) (6.4%), centrally acting agonists (4.3%), atomoxetine (3.0%), mood stabilizers (1.6%) and anxiolytics (1.1%). The most common psychiatric comorbidities were anxiety disorder (7.9%), adjustment disorder (7.8%), disruptive behavior disorders (7.6%), and depressive disorder (6.5%) (Table 1).

**Table 1:** Baseline characteristics of commercially insured youth who initiated stimulants, 2006–2015^a^ (*N* = 61,438).

**Demographic and clinical factors**	***N***	**%**
**Demographic characteristics**		
Age group (years)		
5–9	25,078	40.8
10–14	23,054	37.5
15–17	13,306	21.7
**Sex**		
Female	19,536	31.8
Male	41,902	68.2
**Region**		
East	13,805	22.5
Middle West	18,374	29.9
South	23,212	37.8
West	6,047	9.8
**Psychotropic use**		
Stimulants		
Amphetamine	19,769	32.2
Methylphenidate	41,669	67.8
Atomoxetine	1,815	3.0
Centrally acting agonists	2,613	4.3
**Antidepressants^b^**		
SSRI	3,901	6.4
SNRI	179	0.3
TCA	268	0.4
Anxiolytics	645	1.1
Mood Stabilizers	981	1.6
**Psychiatric Diagnoses**		
Attention deficit hyperactivity disorder	42,057	68.5
Development disorders	1,588	2.6
Schizophrenia	92	0.2
Tic disorder	362	0.6
Bipolar disorder	417	0.7
Disruptive behavior disorders	4,670	7.6
Depressive disorder	4,004	6.5
Anxiety disorder	4,832	7.9
Adjustment disorder	4,782	7.8
Communication and learning disorder	2,129	3.5
Alcohol and other substance abuse	436	0.7
Other psychiatric disorders	3,770	6.1

a *Data source: IQVIA PharMetrics^®^ Plus, January 2006–December 2015*.

b *SSRIs are selective serotonin reuptake inhibitors. SNRIs are serotonin and norepinephrine reuptake inhibitors. TCAs are tricyclic antidepressants*.

### Incidence Rates of Severe and Less Severe Cardiovascular Events

In total, there were 1,096 cardiovascular events (1,064 less severe and 32 severe) over 1,809,861 person-months of follow-up (24,257 current concomitant use, 27,917 past concomitant use, and 1,757,687 no concomitant use). All severe cardiovascular events occurred in person-months with no concomitant and the incidence rate was 0.18 per 10,000 person-months. The incidence rate for less severe cardiovascular events was 14.02 per 10,000 person-months for current concomitant use, 8.24 per 10,000 person-months for past concomitant use, and 5.73 per 10,000 person-months for no concomitant use.

### Cardiovascular Risk and Concomitant Stimulant/AAP Use

Due to the lack of positivity for severe cardiovascular events across concomitant use groups, the analysis was limited to less severe cardiovascular events. In the primary analysis, current concomitant stimulant/AAP use was associated with a significantly increased risk of less severe cardiovascular events compared with no concomitant use [HR:2.59 (95%CI: 1.72, 3.90)] and with past concomitant use [HR: 1.89 (95%CI: 1.10, 3.24)]. Past concomitant use was not significantly associated with increased risk of less severe cardiovascular events compared with no concomitant use [HR: 1.37 (95%CI: 0.89, 2.12)] (Table 2).

**Table 2:** Incidence rates and hazard ratios of less severe cardiovascular risk comparing concomitant use of antipsychotics and stimulants with only stimulant use^a^.

**Status of concomitant use**	**Person-months**	**Cases**	**Incidence rate (per 10,000 person months)**	**Adjusted hazard ratio**	**95% CI**
No concomitant use^b^	1,757,687	1,007	5.73	1.00	ref
Past concomitant use^b^	27,917	23	8.24	1.37^c^	(0.89, 2.12)
Current concomitant use^b^	24,257	34	14.02	2.59^c^	(1.72, 3.90)
				1.89^d^	(1.10, 3.24)
**Current concomitant use by AAP dose^e^**					
Average daily dose of AAP					
<1 mg/day	12,353	14	11.33	2.82^c^	(1.72, 4.61)
1–2 mg/day	6,087	9	14.79	2.22^c^	(1.16, 4.25)
>2 mg/day	5,817	11	18.91	2.65^c^	(1.50, 4.71)
**Current concomitant use by duration of use^f^**					
Cumulative days of concomitant use					
<3 months	8,418	13	15.44	3.45^c^	(2.17, 5.47)
3–6 months	4,355	5	11.48	2.60^c^	(1.29, 5.25)
>6 months	11,484	16	13.93	2.61^c^	(1.59, 4.30)

a *Data source: IQVIA PharMetrics® Plus, January 2006–December 2015*.

b *The model was adjusted for AAP daily dose, duration of AAP use, and DRS*.

c *Compared with no concomitant use*.

d *Compared with past concomitant use*.

e *The model was adjusted for exposure status (no, past, or current concomitant use), duration of AAP use, and DRS*.

f *The model was adjusted for exposure status (concomitant use or not), average daily dose of AAP, and DRS*.

The secondary analysis evaluated the association between the risk of less severe cardiovascular events and (1) average daily AAPs dose of current concomitant use, and (2) duration of current concomitant use. Compared with no concomitant use, the average AAP daily dose (<1, 1–2, and >2 mg/day) of current concomitant use were associated with increased risk of less severe cardiovascular events with no apparent dose response relationship [HR (95% CI): <1 mg/day: 2.82 (1.72, 4.61); 1–2 mg/day: 2.22 (1.16, 4.25); >2 mg/day: 2.65 (1.50, 4.71)]. Relative to no concomitant use, the risk of a less severe cardiovascular event increased across all durations of current concomitant use [HR (95% CI): <3 months: 3.45 (2.17, 5.47); 3–6 months: 2.60 (1.29, 5.25); >6 months: 2.61 (1.59, 4.30)]. The risk of less severe cardiovascular events was highest among youth with current stimulant/AAP concomitant use <3 months. The risk decreased slightly with longer duration of use but remained significant (Table 2).

The sensitivity analysis which allowed only 1 month of loss in health insurance coverage generated similar results as the primary analyses with two exceptions. First, the risk of less severe cardiovascular events comparing current with past concomitant use was not significant [HR (95% CI): 1.52 (0.88, 2.63)]. Second, the risk of less severe cardiovascular events among youth prescribed 1–2 mg AAP daily dose of current concomitant use relative to no concomitant use was not significant [HR (95% CI): 1.90 (0.97, 3.74)].

## Discussion

In a cohort of commercially-insured US youth aged 5–17 years old who were stimulant new users, the incidence rate of severe cardiovascular events was rare. We found a significantly higher risk of less severe cardiovascular events among youth with current concomitant stimulant/AAP use compared with no concomitant use and past concomitant use. We did not observe a significant dose or duration response relationship between AAP dose or duration and the risk of less severe cardiovascular events.

The finding of rare severe cardiovascular events among youth stimulant users is consistent with previous studies (1, 6, 31), however, the significantly increased risk of less severe cardiovascular events related to current concomitant stimulant/AAP use differs from a previous study (30). To the best of our knowledge, this is the only published population-based study that examined the association between concomitant stimulant/AAP use in a cohort of youth who were new stimulant users and the investigators did not find a statistically significant increased cardiovascular risk (30). The differences in findings between our study and the previously published study might be explained by differences in the study design. First, investigators in the prior study defined stimulant/AAP concomitant use as more than 14 days of same day stimulant and AAP use. Our definition examined concomitant use as a time-varying exposure which enabled us to distinguish changes in the regimen over time. Our findings suggest differing risk of less severe cardiac events for current and past concomitant use, which implies a transient risk that may diminish upon discontinuation of concomitant stimulant/AAP use. It is also possible that concomitant use was stopped among youth who showed signs of cardiovascular complications which might explain the lower risk of less severe cardiac event related to past concomitant use relative to current concomitant use. Second, the length of follow-up differed in our study from the previously published study. Instead of focusing on the risk of cardiovascular events within 1 year following stimulant initiation, our study utilized information over a 10-year period. Half of the youth in our cohort had 11 months or more of follow up. This enabled our study to account for the long-term risk of incident less severe cardiovascular events.

We did not observe a dose-response relationship between AAP dose and the risk of less severe cardiovascular events. Other investigators have reported a higher risk of cardiovascular events with increasing AAP dose (13, 20, 37), however the doses reported in these studies were much higher than those observed in our cohort. For example, a study based on Medicaid-insured children observed a 2-fold higher risk of incident cardiovascular events with an AAP daily dose of 3.75 mg or more (risperidone equivalent) compared with 1.25 mg or less (13). In our study, the majority of youth who received concomitant stimulants and AAPs were prescribed AAPs <2 mg per day (risperidone equivalent). The narrow range of average daily dose of AAPs in our study may have limited our ability to detect an AAP dose-response for cardiovascular risk. On the other hand, our findings suggest that concomitant use of stimulants with even low dose of AAP (e.g., <1 mg per day) can increase the risk of developing cardiovascular events among youth. Our study also found that the risk of less severe cardiovascular events was highest in stimulant/AAP concomitant use <3 months, which indicates that adverse cardiovascular events are observed early in the course of the treatment. The risk remained significant with longer duration of use, but lower than that in the first 3 months. It is possible that youth who were least tolerant developed cardiovascular events early in the course of treatment than those who had a longer duration of use. It is also possible that youth may adapt physiologically to the medication over the course of treatment, and thus the cardiovascular risk decreased over time (38).

Our study has several strengths. First, this work adds to the limited evidence of cardiovascular safety related to concomitant use of stimulants and AAPs among youth in the U.S. Second, this is the first study to investigate the cardiovascular safety of AAP dose and duration when prescribed concomitantly with stimulants among youth. Third, we applied a new user design to mitigate prevalent user bias. Fourth, the time-varying approach to define concomitant use accounted for changes in treatment during the follow-up. Nonetheless, this study is not without limitations. Cardiovascular events are rare among youth and thus we had small numbers (i.e., <10) of events for certain subgroups of concomitant stimulant/AAP use, which led to wide confidence intervals of estimated hazard ratios. This may indicate limited precision in risk estimate for these groups. Although a DRS was constructed to adjust for baseline confounders, potential time-varying covariates, including incident physical and psychiatric diagnoses during follow-up, were not considered. Unmeasured confounders may remain as claims data only captures billable health service use and prescribed medications. Therefore, we could not measure use of over-the-counter medications or other potential confounders such as family history, lifestyle, and socioeconomic status. We defined medication use based on prescriptions dispensed in outpatient pharmacies which may not reflect the actual consumption. Our definition of loss to follow-up may lead to missingness due to uninsured months. The sensitivity analysis using a definition that minimized the number of uninsured months showed that the impact of missingness on risk estimates are minimum. Finally, the study may not generalize to US youth who are uninsured or insured through Medicaid, even so the cohort is representative of commercially insured youth.

## Conclusion

Although the incidence of severe cardiovascular events is rare, concomitant stimulant/AAP use is associated with an increased risk of less severe cardiovascular events, including angina pectoris, cardiac dysrhythmias, transient cerebral ischemia, hypertensive disease, tachycardia, palpitations and syncope, among youth stimulant users. The recommendation of periodic monitoring of heart rate and blood pressure may be warranted for youth whose stimulant treatment is augmented to AAPs.

## Data Availability Statement

The data analyzed in this study was obtained from IQVIA. Requests to access these datasets should be directed to https://www.iqvia.com/.

## Author Contributions

CZ: had full access to all of the data in the study and takes responsibility for the integrity of the data and the accuracy of the data analysis, drafting of the manuscript, and statistical analysis. CZ and SD: concept and design. SD and GR: supervision. All authors: acquisition, analysis, or interpretation of data and critical revision of the manuscript for important intellectual content.

## Conflict of Interest

CZ is a Maryland Center of Excellence in Regulatory Science and Innovation (CERSI) Scholar with the US Food and Drug Administration. SD received grant funding from the National Institute of Mental Health (NIMH), the Patient Centered Outcomes Research Institute (PCORI), GSK, and the Pharmaceutical Research Manufacturers of America (PhRMA) Foundation. O'MS was a recipient of a Maryland CERSI Scholar award from the Food and Drug Administration and is currently a PhRMA Foundation pre-doctoral fellow in health outcomes. GR received grant funding from NIMH and PCORI.

## References

[1] Olfson M Huang C Gerhard T Winterstein AG Crystal S Allison PD. Stimulants and cardiovascular events in youth with attention-deficit/hyperactivity disorder. J Am Acad Child Adolesc Psychiatry. (2012) 51:147–56. 10.1016/j.jaac.2011.11.008 22265361 PMC3266532

[2] Jensen PS. A 14-month randomized clinical trial of treatment strategies for attention-deficit/hyperactivity disorder. Arch Gen Psychiatry. (1999) 56:1073–86. 10.1001/archpsyc.56.12.1073 10591283

[3] National Institute of Mental Health Multimodal Treatment Study of ADHD follow-up: 24-month outcomes of treatment strategies for attention-deficit/hyperactivity disorder. Pediatrics. (2004) 113:754–61. 10.1542/peds.113.4.754 15060224

[4] Cooper WO Habel LA Sox CM Chan KA Arbogast PG Cheetham TC. ADHD drugs and serious cardiovascular events in children and young adults. N Engl J Med. (2011) 365:1896–904. 10.1056/NEJMoa1110212 22043968 PMC4943074

[5] Zito JM Burcu M. Stimulants and pediatric cardiovascular risk. J Child Adolesc Psychopharmacol. (2017) 27:538–45. 10.1089/cap.2015.0239 27258470

[6] Winterstein AG Gerhard T Shuster J Johnson M Zito JM Saidi A. Cardiac safety of central nervous system stimulants in children and adolescents with attention-deficit/hyperactivity disorder. Pediatrics. (2007) 120:e1494–501. 10.1542/peds.2007-0675 18055666

[7] Dalsgaard S Kvist AP Leckman JF Nielsen HS Simonsen M. Cardiovascular safety of stimulants in children with attention-deficit/hyperactivity disorder: a nationwide prospective cohort study. J Child Adolesc Psychopharmacol. (2014) 24:302–10. 10.1089/cap.2014.0020 24956171 PMC4137345

[8] Research C for DE and. FDA Drug Safety Communication: Safety Review Update of Medications Used to Treat Attention-Deficit/Hyperactivity Disorder (ADHD) in Children and Young Adults. FDA (2019). Available from: https://www.fda.gov/drugs/drug-safety-and-availability/fda-drug-safety-communication-safety-review-update-medications-used-treat-attention (accessed June 30, 2020).

[9] Winterstein AG Soria-Saucedo R Gerhard T Correll CU Olfson M. Differential risk of increasing psychotropic polypharmacy use in children diagnosed with ADHD as preschoolers. J Clin Psychiatry. (2017) 78:e744–81. 10.4088/JCP.16m10884 28686819

[10] Crystal S Olfson M Huang C Pincus H Gerhard T. Broadened use of atypical antipsychotics: safety, effectiveness, and policy challenges. Health Affairs. (2009) 28:w770–81. 10.1377/hlthaff.28.5.w770 19622537 PMC2896705

[11] Fegert Joerg M Safer Daniel J Kratochvil Christopher J Derivan Albert T Zito Julie M Greenhill Laurence L. Off-label psychopharmacologic prescribing for children: history supports close clinical monitoring. Child Adolesc Psychiatry Mental Health. (2008) 2:24. 10.1186/1753-2000-2-24 18793403 PMC2566553

[12] Maglione M Maher AR Hu J Wang Z Shanman R Shekelle PG. Off-Label Use of Atypical Antipsychotics: An Update. (2011). Available from: https://www.ncbi.nlm.nih.gov/books/NBK66081/ (accessed June 30, 2020). 22132426

[13] Burcu M Zito JM Safer DJ Magder LS DosReis S Shaya FT. Cardiovascular events following treatment initiation with atypical antipsychotic medications in publicly insured U.S. Youth. J Child Adolesc Psychopharmacol. (2018) 28:445–53. 10.1089/cap.2017.0121 29975555

[14] Muscatello MR Spina E Bandelow B Baldwin DS. Clinically relevant drug interactions in anxiety disorders. Hum Psychopharmacol Clin Exp. (2012) 27:239–53. 10.1002/hup.2217 22311403

[15] Schellander R Donnerer J. Antidepressants: clinically relevant drug interactions to be considered. Pharmacology. (2010) 86:203–15. 10.1159/000319744 20829645

[16] Yanofski J. The dopamine dilemma: using stimulants and antipsychotics concurrently. Innovat Clin Neurosci. (2010) 7:18–23. 20622942 PMC2898838

[17] Kreider AR Matone M Bellonci C DosReis S Feudtner C Huang Y-S. Growth in the concurrent use of antipsychotics with other psychotropic medications in medicaid-enrolled children. J Am Acad Child Adolesc Psychiatry. (2014) 53:960–70. 10.1016/j.jaac.2014.05.010 25151419

[18] Comer JS Olfson M Mojtabai R. National trends in child and adolescent psychotropic polypharmacy in office-based practice, 1996-2007. J Am Acad Child Adolesc Psychiatry. (2010) 49:1001–10. 10.1016/j.jaac.2010.07.007 20855045 PMC2952543

[19] Barner JC Khoza S Oladapo A. ADHD medication use, adherence, persistence and cost among Texas medicaid children. Curr Med Res Opin. (2011) 27(Suppl. 2):13–22. 10.1185/03007995.2011.603303 21973228

[20] Ray WA Meredith S Thapa PB Meador KG Hall K Murray KT. Antipsychotics and the risk of sudden cardiac death. Arch Gen Psychiatry. (2001) 58:1161–7. 10.1001/archpsyc.58.12.1161 11735845

[21] Cleophas TJ. Carryover bias in clinical investigations. J Clin Pharmacol. (1993) 33:799–804. 10.1002/j.1552-4604.1993.tb01954.x 8227475

[22] Burcu M Zito JM Safer DJ Magder LS dosReis S Shaya FT. New research: concomitant use of atypical antipsychotics with other psychotropic medication classes and the risk of type 2 diabetes mellitus. J Am Acad Child Adolesc Psychiatry. (2017) 56:642–51. 10.1016/j.jaac.2017.04.004 28735693

[23] Sharp WS Walter JM Marsh WL Ritchie GF Hamburger SD Castellanos FX. ADHD in girls: clinical comparability of a research sample. J Am Acad Child Adolesc Psychiatry. (1999) 38:40–7. 10.1097/00004583-199901000-00018 9893415

[24] Vitiello B. Methylphenidate in the treatment of children with attention-deficit hyperactivity disorder. Can Med Assoc J. (2001) 165:1505. Retrieved from: https://www.cmaj.ca/content/165/11/1505.long (accessed June 30, 2020). 11762576 PMC81668

[25] Goldstein LB. Accuracy of ICD-9-CM coding for the identification of patients with acute ischemic stroke: effect of modifier codes. Stroke. (1998) 29:1602–4. 10.1161/01.STR.29.8.1602 9707200

[26] Kiyota Y Schneeweiss S Glynn RJ Cannuscio CC Avorn J Solomon DH. Clinical investigations: accuracy of medicare claims-based diagnosis of acute myocardial infarction: estimating positive predictive value on the basis of review of hospital records. Am Heart J. (2004) 148:99–104. 10.1016/j.ahj.2004.02.013 15215798

[27] Rosamond WD Chambless LE Sorlie PD Bell EM Weitzman S Smith JC. Trends in the sensitivity, positive predictive value, false-positive rate, and comparability ratio of hospital discharge diagnosis codes for acute myocardial infarction in four US communities, 1987-2000. Am J Epidemiol. (2004) 160:1137–46. 10.1093/aje/kwh341 15583364

[28] Tirschwell DL Longstreth WTJ. Validating administrative data in stroke research. Stroke. (2002) 33:2465–70. 10.1161/01.STR.0000032240.28636.BD 12364739

[29] Heckbert SR Kooperberg C Safford MM Psaty BM Hsia J McTiernan A. Comparison of self-report, hospital discharge codes, and adjudication of cardiovascular events in the Women's Health Initiative. Am J Epidemiol. (2004) 160:1152–8. 10.1093/aje/kwh314 15583367

[30] Bali V Kamble PS Aparasu RR. Cardiovascular safety of concomitant use of atypical antipsychotics and long-acting stimulants in children and adolescents with ADHD. J Atten Disord. (2015) 23:163–72. 10.1177/1087054715608443 26494504

[31] Winterstein AG Gerhard T Shuster J Saidi A. Cardiac safety of methylphenidate versus amphetamine salts in the treatment of ADHD. Pediatrics. (2009) 124:e75–80. 10.1542/peds.2008-3138 19564272 PMC3856396

[32] Miettinen OS. Stratification by a multivariate confounder score. Am J Epidemiol. (1976) 104:609. 10.1093/oxfordjournals.aje.a112339 998608

[33] Glynn RJ Gagne JJ Schneeweiss S. Role of disease risk scores in comparative effectiveness research with emerging therapies. Pharmacoepidemiol Drug Safety. (2012) 21(Suppl. 2):138–47. 10.1002/pds.3231 22552989 PMC3454457

[34] Arbogast PG Ray WA. Use of disease risk scores in pharmacoepidemiologic studies. Stat Methods Med Res. (2009) 18:67–80. 10.1177/0962280208092347 18562398

[35] Hansen BB. The prognostic analogue of the propensity score. Biometrika. (2008) 95:481. 10.1093/biomet/asn004

[36] Arbogast PG Ray WA. Performance of disease risk scores, propensity scores, and traditional multivariable outcome regression in the presence of multiple confounders. Am J Epidemiol. (2011) 174:613–20. 10.1093/aje/kwr143 21749976

[37] Lin S-T Chen C-C Tsang H-Y Lee C-S Yang P Cheng K-D. Association between antipsychotic use and risk of acute myocardial infarction: a nationwide case-crossover study. Circulation. (2014) 130:235–43. 10.1161/CIRCULATIONAHA.114.008779 24838361

[38] Miettinen OS Caro JJ. Principles of nonexperimental assessment of excess risk, with special reference to adverse drug reactions. J Clin Epidemiol. (1989) 42:325–31. 10.1016/0895-4356(89)90037-1 2723693

