# Supplementary material for: Cardiovascular Risk of Concomitant Use of Atypical Antipsychotics and Stimulants Among Commercially Insured Youth in the United States
Source: Front Psychiatry. 2021 Apr 29;12:640244. doi: 10.3389/fpsyt.2021.640244 (PMC8116622; doi:10.3389/fpsyt.2021.640244)
Supplement: Supplementary file 1 [file Table_1.DOCX]

| **Appendix A. Covariate List and ICD-9-CM Diagnosis codes, CPT-4 procedure codes, the Generic Product Identifier and Generic Names Used** | | | | |
| --- | --- | --- | --- | --- |
| Covariates | ICD-9-CM^a^ | CPT -4^b^ | GPI^c^ | Generic names |
| Schizophrenia/other psychoses | 295, 297, 298 |  |  |  |
| Pervasive development disorder/intellectual disability | 299, 317-319 |  |  |  |
| Tic disorder | 307.2 |  |  |  |
| Bipolar disorder | 296.0, 296.1, 296.4-296.7, 296.80, 296.89, 301.13 |  |  |  |
| Disruptive behavior disorder | 312.0-312.4, 312.81, 312.82, 312.89, 312.9, 313.81 |  |  |  |
| Attention-deficit/hyperactivity disorder | 314 |  |  |  |
| Depressive disorder | 293.83, 296.2, 296.3, 296.9, 298.0, 300.4, 311 |  |  |  |
| Anxiety disorder | 293.84, 300.0, 300.2, 300.3, 308.3, 309.21, 309.81, 313.0, 313.2, 313.89 |  |  |  |
| Adjustment disorder | 308.0-308.2, 308.4, 308.9, 309.0-309.4, 309.82, 309.83, 309.89, 309.9 |  |  |  |
| Communication and learning disorder | 307.0, 307.9, 315.0-315.2, 315.31, 315.32, 315.39, 315.9 |  |  |  |
| Alcohol and other substance use disorder | 291, 292, 303, 304, 305, V1.13 |  |  |  |
| Other psychiatric disorders | V40,V66.3,V67.3,290,294,295,299,302,303,304,305,306,310,316,317,318,319 |  |  |  |
| Overweight/obese | 278.0, 783.1, V65.3, V77.8, V85.3, V85.4, V85.53, V85.54 | 97802, 97803 |  |  |
| Hyperlipidemia | 272.0-272.2, 272.4 |  |  |  |
| Thyroid related disorders | 240-246 |  |  |  |
| Diabetes | 250 |  |  |  |
| Other endocrine and metabolic disorder | 251-259, 270, 271, 272.3, 272.5-272.9, 275.0-275.3, 277 |  |  |  |
| Lipid test |  | 80061, 82465, 83700, 83701, 83715, 83716, 83721, 84478, 83718 |  |  |
| Glucose test |  | 82947, 82948, 82950, 82951, 82952, 83036, 82962 |  |  |
| Congenital anomalies of heart and circulatory system | 745-747 |  |  |  |
| Renal insufficiency | 582-588 |  |  |  |
| Cardiovascular symptoms | 780.2, 785.0, 785.1, 785.2, 785.3, 785.50, 785.51, 785.9, 786.5 |  |  |  |
| Asthma | 493 |  |  |  |
| Other respiratory disease and related conditions | 490-492, 494-496 |  |  |  |
| Smoking (diagnosed) | 305.1, 649.0, 989.84, V15.82 | 99406, 99407 |  |  |
| Other respiratory symptoms | 995.0, 995.27, 995.29, 995.3, 995.6, 786.05, 786.07, 799.0  786.00-786.04, 786.06, 786.09, 786.1-786.4, 786.8, 786.9 |  |  |  |
| Antidepressants |  |  |  | Fluoxetine, Fluvoxamine, Paroxetine, Sertraline, Vilazodone, Duloxetine, Levomilnacipran, Venlafaxine, Amoxapine, Desipramine, Doxepin , Imipramine, Nortriptyline, Protriptyline, Trimipramine, Phenelzine, Selegiline , Tranylcypromine |
| Anxiolytics |  |  | 5700000000 |  |
| Mood stabilizers |  |  |  | Carbamazepine, Divalproex, Lamotrigine, Gabapentin, Oxcarbazepine, Topiramate, Valproate, Valproic |
| Atomoxetine |  |  |  | Atomoxetine |
| Central Active Agonist |  |  |  | Clonidine, Guanfacine |
| Cardiovascular medications |  |  | 3120000000 (Cardiac glycosides), 3720000000 (Loop diuretics), 3760000000 (Thiazides and thiazide-like diuretics), 3510000000 – 3550000000 (Antiarrhythmics), 3320000000 (Cardio-selective beta blockers), 3400000000 (Calcium channel blockers) | Benazepril, Captopril, Ntinued, Enalapril, Fosinopril, Lisinopril, Moexipril, Perindopril, Quinapril, Ramipril |
| Oral contraceptive/devices |  |  |  | Desogestrel, Drospirenone, Ethinyl, Estradiol, Ethynodiol, Diacetate,  Levonorgestrel, Mestranol, Norethindrone, Norgestimate, Norgestrel, Diaphragm, Norethindrone, Etonogestrel,  Intra-uterine device, Levonorgestrel, Nonoxynol, Norelgestromin, Medroxyprogesterone |

1. ICD-9-CM codes: International Classification of Diseases, Ninth Revision, Clinical Modification
2. CPT-4: Current Procedure Terminology
3. GPI: The Generic Product Identifier
